# Supportive care needs of adults living with a peripherally inserted central catheter (PICC) at home: a qualitative content analysis

**DOI:** 10.1186/s12912-023-01614-0

**Published:** 2024-01-02

**Authors:** Rebecca Sharp, Qunyan Xu, ‬‬‬‬‬‬‬‬‬‬‬Robyn Pumpa, Lisa Elliott, Nadia Corsini, Julie Marker, Jodie Altschwager, Alanna Ortmann, Lisa Turner, Lili Jin, Amanda Ullman, Adrian Esterman

**Affiliations:** 1 Clinical & Health Sciences, University of South Australia, Adelaide, Australia; 2 Royal Adelaide Hospital, Central Adelaide Local Health Network, Adelaide, Australia; 3 Rosemary Bryant AO Research Centre, University of South Australia, Adelaide, Australia; 4 Cancer Voices South Australia, Adelaide, Australia; 5 Metropolitan Referral Unit, SA Health, Adelaide, Australia; 6 Silver Chain, Adelaide, Australia; 7 South Australia Medical Imaging (SAMI)/ Royal Adelaide Hospital, Central Adelaide Local Health Network, Adelaide, Australia; 8 School of Nursing, Midwifery and Social Work, University of Queensland, Brisbane, Australia; 9 Allied Health & Human Performance, University of South Australia, Adelaide, Australia; 10 Children’s Health Queensland Hospital and Health Service, Brisbane, QLD Australia

**Keywords:** Supportive care needs, Peripherally inserted central catheter (PICC), Outpatient parenteral antimicrobial therapy (OPAT), Chemotherapy, Hospital in the Home (HITH)

## Abstract

**Background:**

Peripherally inserted central catheters (PICCs) are common vascular access devices inserted for adults undergoing intravenous treatment in the community setting. Individuals with a PICC report challenges understanding information and adapting to the device both practically and psychologically at home. There is a lack of research investigating the supportive care needs of individuals with a PICC to inform nursing assessment and the provision of additional supports they may require to successfully adapt to life with a PICC. The aim of this study was to identify the supportive care needs of adults with cancer or infection living with a PICC at home.

**Method:**

Qualitative, semi-structured interviews were used to identify supportive care needs of adults living with a PICC at home. Participants were recruited from cancer and infectious diseases outpatient units. Two researchers independently analysed transcripts using content analysis.

**Results:**

A total of 15 participants were interviewed (30–87 years old). There were 5 males and 10 females interviewed, 9 participants had a cancer diagnosis and most lived in a metropolitan area. Many participants lived with a partner/spouse at home and three participants had young children. Participants identified supportive care needs in the following eight categories (i (i) Adapting daily life (ii) Physical comfort (iii) Self-management (iv) Emotional impact (v) Information content (vi) Understanding information (vii) Healthcare resources and (viii) Social supports.

**Conclusions:**

Adults living with a PICC at home report a broad range of supportive care needs. In addition to practical and information needs, health consumers may also require support to accept living with a device inside their body and to assume responsibility for the PICC. These findings may provide nurses with a greater understanding of individual needs and guide the provision of appropriate supports.

**Supplementary Information:**

The online version contains supplementary material available at 10.1186/s12912-023-01614-0.

## Background

Intravenous treatment in the community provided through clinical models including home nursing programs and outpatient infusion centres has grown markedly in the last decade, with the recent COVID 19 pandemic further increasing demand [1]. Peripherally inserted central catheters (PICCs) are medium to long-term vascular access devices recommended for consumers undergoing intravenous treatment at home or outpatient facilities [2]. These devices are commonly used to facilitate community treatment for those with serious infection and cancer [3] who are the most common diagnostic groups who receive a PICC [4–6] Under these clinical models, health consumers have a PICC inserted and receive treatment from a nurse at home or attend an outpatient infusion centre. Regardless of the clinical model, individuals with a PICC are required to make major adjustments to daily life and assume increased responsibility for their care as they have reduced contact with clinical staff when compared to individuals in hospital [7, 8].

Despite having different diagnoses, individuals undergoing treatment for serious infection and cancer with a PICC in the community have reported similar practical, social, informational and psychological challenges [8]. Both cohorts have reported that adapting usual tasks, such as showering, to prevent complications [9–14]. Similarly, individuals receiving treatment for cancer and infection with a PICC have indicated that they felt underprepared for life with the device and described challenges understanding PICC information [11, 12, 15]. Research in both cohorts has found that some people face psychological challenges with accepting the PICC, as well as perceived stigma from the device, resulting in a need to conceal the PICC from view [10, 16, 17]. Some avoid social activities and reduce usual interactions with their children, which impacts their quality of life [10, 13].

Health consumers in both groups have expressed fear of vascular access device-associated adverse events at home [13, 18–22] Individuals are required to take increased responsibility as they self-manage these complex devices without a clinician present for most of the time. This includes identifying and initiating clinical care for PICC complications. PICC complication rates of consumers undergoing treatment for infection and cancer in the community are similar to those admitted to acute care facilities (6–25%) [3, 23–26]. Unsurprisingly, they report anxiety about the risk of PICC associated complications [12, 13, 19, 20, 22]. This distress is amplified as there is an expectation to identify/escalate PICC complications independently [13].

Supportive care refers to the supports and services people require to successfully adjust to a health condition and continue daily life [27]. This approach is person-centred and seeks to identify needs and provide interventions to reduce health condition symptoms, improve understanding, maximise existing functional ability, and promote adaptation [28]. A framework to assist clinicians to assess supportive care needs in people undergoing cancer treatment was first conceived by Fitch in the 1990s [27]. The supportive care needs framework categorises needs into physical, emotional, social, psychological, informational, spiritual, health care, and practical care domains. This framework has informed the assessment of needs in a broad range of health conditions including adults with colon cancer [29] those experiencing first episode psychosis [30] and families of children with rare diseases [31].

Despite the challenges that individuals face to successfully adapt to life with a PICC, there is a lack of research investigating their supportive care needs [8]. One study examined the supportive care needs of adults with a PICC receiving treatment for infection in the community [11]. However, they focussed on their perception of the Outpatient parenteral antimicrobial therapy (OPAT) clinical model and needs for system change to improve patient-centred care, rather than the specific and personal needs of consumers living with a PICC. There is a plethora of studies that have examined the supportive care needs of people with cancer generally, and according to individual factors such as age [32] and specific cancer type [33, 34]. The above research has not specifically investigated needs relating to the vascular access device (VAD) used to provide treatment (PICC or other VAD).

Individual studies indicate that adults living with a PICC in the community may have unmet needs, which impacts daily life and affects their ability to safely participate in this model of care [8]. Yet, as far as we are aware, a study has yet to be conducted to explore and document the supportive care needs of individuals with a PICC living at home. Nurses may not appreciate the supportive care needs of adults living with a PICC. An improved understanding of consumer needs may facilitate nursing assessment and the provision of additional supports they require to successfully adapt to life with a PICC. This would also allow the provision of person-centred care whose underlying tenet is to provide healthcare that is responsive to individual needs [35]. The aim of this study was to identify the supportive care needs of adults with cancer or infection living with a PICC at home.

## Method

### Study design

A qualitative approach using semi-structured interviews was used to identify the needs of adults with a PICC at a tertiary hospital in Adelaide, South Australia. Specifically, qualitative description was used, an approach that seeks to describe the perspective of participants and stay ‘close to the data’ rather than interpret responses from a conceptual framework [36]. The project is reported in accordance with the EQUATOR network consolidated criteria for reporting qualitative research (COREQ) [37]. Supportive care needs were identified when participants described challenges continuing daily life that were associated with the PICC or identified that supports were required to maintain usual functioning.

An interview guide (Table 1) was developed based on a scoping review about the experience and supportive care needs of individuals undergoing intravenous treatment in the community [8] and published literature about supportive care needs including Fitch’s model of supportive care needs in individuals with cancer [27, 30, 31] The questions related to five key supportive care need areas: social, physical, information, emotional and practical domains. Participants were also asked to identify any other challenges or supports they required to determine if any needs sat outside these areas.

**Table 1:** Interview guide

**Overall experience**
1. Tell me about why you needed a peripherally inserted central catheter (PICC)
2. What was the insertion like?
3. Tell me about how living with a PICC was for you
4. How were the first 24 h after you had the PICC inserted?
5. After you got used to living with the PICC, tell me about what a normal day looked like living with a PICC e.g. from waking up
6. What were the daily problems you faced?
7. Did you get much help for these problems from doctors or nurses?
**Physical**
8. Did you have any pain after the PICC was put in?
9. What about the dressing, was this comfortable?
**Practical**
10. What did you do to make it easier to live with a PICC?
11. Tell me about how you showered/dressed/slept/worked/exercised with a PICC?
12. Did it take long to get used to living with a PICC?
13. What would help you with this? E.g. more help from nurses
**Information**
14. Tell me about the information you received about the insertion and living with the PICC e.g. showering and looking out for things that might go wrong
15. Did the nurses give you written information, or did they tell you about the insertion and living with the device?
16. Did you find the information useful? Easy to understand?
17. Did you experience fatigue and did this impact on your ability to understand how to manage everything?
18. Did you feel prepared for the PICC insertion/living with a PICC?
19. Do you think most people would understand the information that nurses/doctors give people with a PICC?
20. How do you think we should give information to people about the PICC? E.g. written, 1:1 teaching, group education, online information etc
**Emotional**
21. How did having a PICC affect your life?
22. How did you feel about having the PICC inside your body?
23. Did you have any fears or uncertainties about living with a PICC at home?
24. How did you feel about the risk of things going wrong at home?
25. How did you feel about needing to watch for things going wrong when you were at home?
26. Did you feel that you needed to take more responsibility/ownership for managing the PICC at home?
27. How do you think nurses and doctors could help people to be comfortable in taking responsibility for managing the PICC at home?
**Social**
28. What did other people say about the PICC? Did you try to hide the PICC?
29. Did you go out much with the PICC?
30. Did the PICC affect how you were around other people – family and friends?
**General**
31. Looking back, what were your greatest needs during this time? Could you list your needs?
32. What do you think are the needs of people living with a PICC?
33. What do you think would help other people living with a PICC?

### Setting

Participants were recruited from a large metropolitan public teaching hospital for adults. The hospital is the main provider of cancer and infectious diseases outpatient treatment in South Australia.

### Clinical model

At the hospital where the study was set, a PICC is inserted in the Radiology department by specialist nurses. Commonly, for those with an infection, treatment is initiated as an in-patient, and they are discharged to community-based intravenous antibiotic treatment. Individuals attend an outpatient department (OPD) infusion centre for medical appointments, PICC care, and daily intravenous antibiotic infusions (common for antibiotics with a long half-life). Alternatively, the consumer is discharged home with a continuous infusion, often under a hospital in the home (HITH) program or supported/early discharge model. In this model, a nurse attends the home to change or disconnect the infusion and provide PICC care. Cancer treatment is typically delivered in an ambulatory setting. Some have their entire infusion in the cancer infusion centre and others attend the centre to have part of their chemotherapy regimen, then they leave with a continuous infusion over several days, which is disconnected or changed by a visiting nurse.

### Recruitment and participants

Ethical approval was obtained prior to the study’s commencement (CALHN: 15,339).

A purposeful sampling approach was used to select participants based on diagnosis types (haematological cancers/solid tumours and infection) and location (metropolitan vs. rural) to ensure a wide range of consumer experiences were captured.

#### Inclusion criteria

- Adults (18 years or older) with cancer or an infection with a PICC
- Living at home or residential care facility
- Access to computer and reliable internet connection
- Willing to participate in a telephone/video interview

#### Exclusion criteria

- Unable to provide informed consent

The Nurse Consultant of the Infectious Diseases Infusion Clinic and Nurse Unit Manager of the Cancer Day Treatment Unit informed individuals of the study. Researchers (RP, LE) approached individuals to take part in the study and provided an information sheet if they were interested. They did not obtain informed consent from participants due to their existing clinical relationship with these individuals as it was thought that this may influence their ability to provide voluntary consent. Potential participants were provided with a flyer with a link to a website where they could access an information sheet and provide consent, or they were given a paper information sheet and consent form and they were consented by a researcher employed by the university (RS).

### Data collection

Semi-structured telephone or video interviews (decided by participants) were conducted by RS from January 2022 to March 2023 and were recorded. An interview guide was used to direct the interviews (Table 1). Participant responses guided which domains were explored further. Participants were asked to validate the researcher’s interpretation of their responses during the interview process to increase accuracy. Interviews were conducted by RS, an adult nurse who has > 10 years clinical experience in the management of PICCs, is a vascular access device researcher with a doctoral degree and has conducted prior qualitative research in the health consumer experience of a PICC.

### Data analysis

Data analysis commenced after all data collection was completed, as the aim of the research was to identify the supportive care needs of individuals with a PICC, rather than develop a theoretical understanding of the phenomena (where an iterative approach would be appropriate). Interviews were transcribed by a professional transcription service. Data from the interviews was analysed as per Graneheim and Lundman’s qualitative content analysis technique [38]. Both manifest (where participants directly stated their needs) and latent content (where researchers inferred needs from the participants’ description of their experience) were analysed. A unit of analysis comprised each transcribed interview. Two researchers (RS and QX) independently read transcripts several times for an overall understanding of the phenomena. Meaning units or groups of words which gave the same meaning or similar concepts were identified and labelled with a code. Two researchers compared coding of all transcripts and any disagreements were discussed until consensus was reached. Similar codes were grouped into sub-categories. Sub-categories were compared with each other and grouped into categories. An example of the analysis is provided in supplementary file 1. It was unknown whether supportive care needs would differ according to diagnosis, hence, once categories were decided, extracted codes and sub-categories were separated according to diagnosis within the category to determine if needs were apparent for both cohorts. Data collection was conducted until data saturation occurred. This was determined when similar concepts were repeated, and no new ideas were provided by participants.

## Results

A total of 15 participants were interviewed and ages ranged from 30–87 years old. One recording failed which was realized immediately after the interview was completed and the researcher made notes of the participant’s responses which were included in the analysis. There were 5 males and 10 females interviewed, 9 participants had a cancer diagnosis and most lived in a metropolitan area. Most participants lived with a partner/spouse and three participants had young children (Table 2). While we aimed to also include those living in residential care facilities, none were recruited. All participants lived in a private residential house, and some from rural areas/ interstate stayed in temporary accommodation for part of their treatment.

**Table 2:** Participant information

	**Age**	**Gender**	**location**	**Diagnosis**	**Previous PICC?**	**Clinical information**	**Living arrangement**
1	87	F	Metro	Outer ear Infection	N	PICC inserted as an in-patient – 1 day with PICC on the ward D/C home with IVAB infusion (elastomeric device). Infusion/dressing changed at home	Lives alone in retirement village
2	71	F	Outer Metro	Bladder Cancer	N	PICC inserted as an outpatient Infusion/dressing at main cancer centre/local hospital	Lives with spouse at home
3	59	M	Metro	Acute myeloid leukaemia	Y –4 previous PICCs	PICC inserted as an outpatient. Hospitalised for a few weeks with a PICC during treatment Infusion/dressing at main cancer centre/local hospital	Lives with spouse at home (interstate). Stayed at an accommodation service for people with cancer from rural areas while undergoing treatment and attended the OPD daily
4	57	M	Rural	Acute myeloid leukaemia	N	PICC inserted as an outpatient. Few weeks with PICC as in-patient Infusion/dressing at main cancer centre/local hospital	Lives with spouse at home Stayed at an accommodation service for people with cancer from rural areas while undergoing treatment and attended the OPD daily
5	45	M	Rural	Sepsis, lung/spine abscess	N	PICC inserted as an in-patient—1 week with a PICC on the ward D/C home with IVAB infusion (elastomeric). Infusion/dressing changed at home	Lives with spouse/children at home
6	51	F	Metro	Jaw osteomyelitis	N	PICC inserted as an outpatient Treatment at home with IVAB infusion (elastomeric). Infusion/dressing changed at home	Lives with spouse at home
7	75	M	Rural	Acute myeloid leukaemia	Y- 3 previous PICCs	PICC inserted as an in-patient—1 week with PICC on the ward Infusion/dressing at main cancer centre/local hospital	Lives with spouse at home
8	78	F	Metro	Abdominal abscess post-surgery	N	PICC inserted as an in-patient – 1 week with PICC on the ward D/C home with IVAB infusion (elastomeric). Infusion/dressing changed at home	Lives with spouse at home
9	68	F	Metro	CMML	N	PICC inserted as an outpatient Infusion/dressing at main cancer centre/local hospital	Lives with spouse at home
10	72	F	Rural	Bowel Cancer	N previous TIVAD	PICC inserted as outpatient Infusion/dressing at main cancer centre/local hospital	Lives alone at home in a rural area with PICC in between treatment. Lived with sister in Adelaide whilst undergoing treatment at OPD
11	68	M	Metro	Multiple myeloma	Y – 1 previous PICC	PICC inserted as an in-patient -1 week with PICC on the ward then D/C home Infusion/dressing at main cancer centre/local hospital	Lives with spouse at home
12	30	F	Metro	Acute myeloid leukaemia	N	PICC inserted as an in-patient—8 weeks with PICC on ward then D/C home Infusion/dressing at main cancer centre/local hospital	Lives with spouse and child at home (toddler)
13	52	F	Metro	Acute myeloid leukaemia	N	PICC inserted as an in-patient—4 weeks with PICC on the ward then D/C home Infusion/dressing at main cancer centre/local hospital	Lives with child at home (toddler)
14	66	F	Metro	Infected hip prothesis	Y – 1 previous PICC	PICC inserted as an in-patient (interstate)—3 weeks with PICC on the ward. D/C to sister’s house with IVAB infusion (elastomeric). Infusion/dressing changed at home	Lives with spouse at home (interstate) Lived with sister in Adelaide whilst undergoing treatment
15	79	F	Metro	Spine osteomyelitis	N	PICC inserted as an in-patient -1 day with PICC on the ward. D/C home with IVAB infusion (elastomeric). Infusion/dressing changed at home	Lives at home alone

**Fig. 1:**
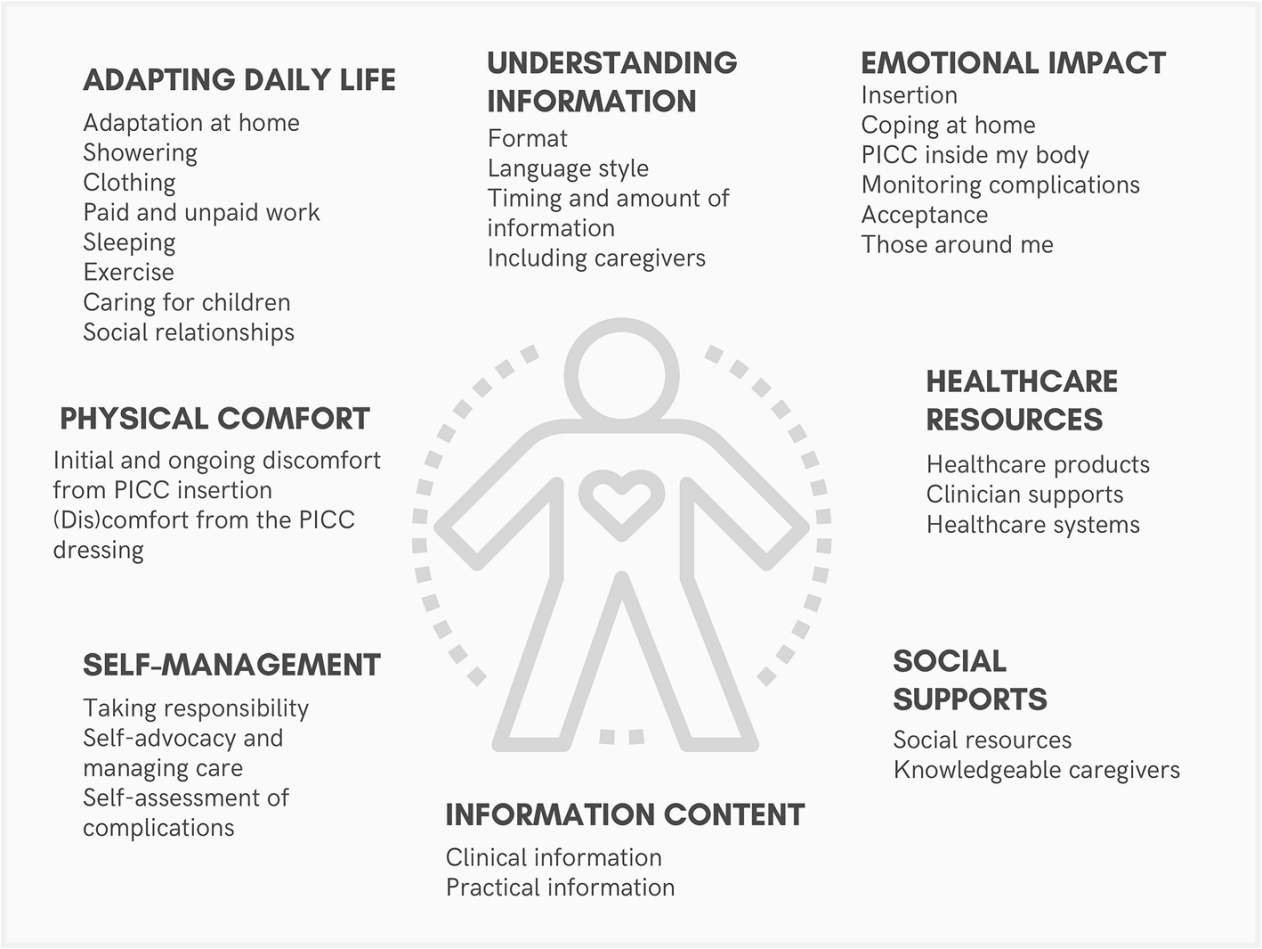
Supportive care needs of adults living with a PICC at home

Participants identified supportive care needs in the following eight main categories (i) Adapting daily life (ii) Physical comfort (iii) Self-management (iv) Emotional impact (v) Information content (vi) Understanding information (vii) Healthcare resources and (viii) Social supports. Both individuals with cancer and infection indicated supportive care needs in all domains. Supportive care need categories and sub-categories are presented in Fig. 1.

### Adapting daily life

Adaptation of daily activities to accommodate the PICC was the most common challenge stated by participants and associated with their greatest needs. Modifying showering to protect the PICC was difficult, and some participants were unable to do this independently. This meant they required informal caregivers or paid nursing staff to provide additional support. While most activities could be modified to protect the PICC, some were too risky, and participants were forced to stop these activities while they had the device.

#### Adaptation at home

Once participants arrived home with a PICC, they described initial uncertainty about the modifications required to their usual activities to protect the PICC. Daily activities were viewed as a threat to the PICC, and they became ‘*wary*’ and ‘*cautious*.’ Many of those who had a PICC while they were hospitalised found this experience assisted with the adaptation process at home. Participants learnt how to manage and accept the PICC through the experience of watching nurses care for the device. Some participants who had leukemia and spent weeks in hospital with the PICC described that they ‘*learnt it all*’ whilst in hospital, and that this experience ‘*trained them’* to live with the device. However, one participant with a young child found their experience in hospital did not aid adaptation and described being at home and hospital as two different experiences. They recounted that their activity in hospital was limited due to their illness. Once they were home they suddenly faced parenting and household responsibilities and were unsure how to adapt these activities so they could continue their usual role. Another participant with infection described that while they felt comfortable in hospital with a PICC, they faced initial uncertainty once the arrived home and indicated that individuals with a PICC cannot predict how well they will adapt until they have experienced life at home for themselves.

‘Like, you don’t know exactly how it’s going to go until you get in the car and drive out the hospital and go, okay, we’re on IV now, 24/7, so let’s see how this goes, and just modify.’ P5

#### Showering

Showering with the PICC was the most common challenge identified by participants. Applying the shower cover was described as ‘inconvenient’ and ‘frustrating’ and many found these were ineffective in keeping the dressing dry. This meant that they were required to modify how they showered. They learnt to keep one arm out of the shower and only use the ‘non-PICC’ arm to wash themselves. This made washing their hair especially difficult as they could only use one hand.

‘…those sleeves tend to leak if you get them wet and water runs off your head and …and down your arm and the next thing you know it’s leaked through but, you know, you learn how to stand like the Statue of Liberty … if you keep the closure end of it or the elastic bit above the level of the water you’re better off, because if you’ve got your arm down by your side it tends to splash off your shoulder or whatever, and the next thing you know it’s leaked and you’ve got your dressing wet.’P7

Participants were aware of the importance of adapting showering to keep the dressing dry to reduce the risk of infection. The risk of infection made some participants anxious about showering and they used more than one shower cover to ensure moisture did not enter the dressing.

‘More about the shower because the first time I just scared, because they said if it get wet, if it got an infection it go to your body, is near to your heart… I was very scared... That’s why I just put two, three plastic shower bags to cover it.’ P12

While most participants were able to successfully modify showering to protect the PICC, some found that they could not do this independently. This was due to both the PICC and their underlying medical condition. Either an informal caregiver such as their spouse or paid nursing staff were required to assist them in the shower at home. These participants identified that it would be difficult to manage applying a shower cover and showering with a PICC alone.

‘…you know, if you were by yourself it would probably be - one of the things that would be hard to manage, you know, in terms of keeping it water tight because, you know, trying to get - get it to a point where no water can seep in. I’ve used plastic sleeves and stuff like that. But, you know, (spouse) helps me with all of that.’ P2

Successful adaptation of showering was a turning point for many participants. They became comfortable living with a PICC at home and accepting of the device once they were able to keep the dressing dry in the shower.

#### Clothing

Changing clothing was difficult with the PICC and some participants required assistance from their spouse for the entire time they had a PICC. Participants also described the need to adapt clothing choice to allow easy access for treatment and to hide the PICC. Clothing with tight sleeves was avoided to allow easy access for treatment, and many participants made modifications to their clothing choice or used the hospital supplied PICC cover to hide the PICC.

‘You need to change your wardrobe though, you need long sleeves with everything because it’s not pleasant sort of, you are walking around with a bandage on your arm, if you wear short sleeved anything.’ P10

Initially, some participants would only wear long-sleeved tops to hide the PICC. Once they accepted the device, they stopped trying to conceal the PICC and the device no longer influenced clothing choice. Many participants were thankful for the black bamboo PICC cover provided by the hospital. This cover allowed participants to discreetly cover the PICC when they wore short-sleeved tops, and they welcomed the design, which was viewed as less ‘medical looking’ than an elasticised bandage.

‘That (elasticised bandage) is a lot more obvious than this. This could be some trendy sweatband.’P13

#### Paid and unpaid work

The PICC had varying impact on the adaptation required for both paid and unpaid work. Many participants described that they were no longer able to complete household duties, such as cleaning, due to fatigue. For those still able to continue these activities, adaptation was required to continue their usual household duties. Participants described that they stopped using the ‘PICC arm’ for activities such as mopping and vacuuming to protect the PICC. This was challenging for many participants, as most had the PICC inserted in their dominant arm.

Those who lived in rural areas described additional challenges with life on a farm with a PICC. Their usual role on the farm often included activities that were a threat to the PICC such as lifting heavy loads and repetitive arm movements. They learnt to be wary about which activities they could continue and use their ‘non-PICC’ arm to continue usual activities such as carrying heavy items. Some participants were unable to make these modifications due to the impact of their underlying diagnosis and the PICC. This meant that their family were required to take responsibility for these activities whilst they had the PICC in place. The challenges in adapting usual activities on a farm was described by one participant as one of their greatest needs.

‘…she was helping, doing all the major chores on the farm, so they were the biggest needs. I couldn’t … I walked around watching my wife do them, or held the carton for the eggs, or you know, I’d just do the tiny bits. .. so I just was there to hold (gates) open and shut, and you know, things that I could handle, but I couldn’t do anything major until my PICC.. was removed, then I could start carrying buckets again and stuff, because I didn’t want to have any adverse effect.’ P5.

Most participants were not in paid employment prior to PICC insertion (due to retirement or caring responsibilities) or stopped work due to the impact of their health condition. Those still in paid employment described different levels of adaptation and supports required to continue their usual duties. One participant continued work as a massage therapist and indicated that they were required to spend time modifying how they provided a massage to protect the PICC.

‘I’m still working for a living and I’m a massage therapist … you have to approach something from a slightly different angle, like you pick up limb that you want to work on and you can’t sort of wrap your arm around it like you used to sort of thing so, you know, you can attack other people from different angles, it’s not a major issue but you just have to think about it.’ P7

Another participant described that they were able to continue their usual occupation unimpeded due to the nature of their working conditions and support from their spouse. They worked from home managing a small business and their role was largely computer based, so they required minimal modifications to continue working whilst undergoing treatment with a PICC.

#### Sleeping

Some participants described that the PICC made sleep challenging. One participant (who did not have an elastomeric device/infusion attached) found that the external part of the PICC (needleless connectors) would get caught in their elbow, which would disturb their sleep. It was only once they requested assistance from the nurses at the OPD that they learnt to tape these parts at night to keep it secure. Other participants described that they initially modified their sleep position to protect the PICC by avoiding sleeping on the side the PICC was inserted, as they assumed this would damage their PICC. However, they soon realised they could not sustain a changed sleep position and began to sleep on their usual side.

‘Yeah, so not moving your arm. And I tend to sleep on that side, so no harm can come to it now. You sort of protect it with your body, I think…it turned out to be my normal side, I sleep on my left hand side and my PICC line is on my left had side, so it’s just tidy. For a while there I was sleeping on my back and worried about it, but once I just relaxed and got back to normal again, I was right.’ P10

#### Exercise

Many participants reported that they had ceased their usual exercise routine while they had a PICC due to the impact of their medical condition. Some participants felt able to continue exercise but were unsure which activities were safe with a PICC and this uncertainty meant they avoided exercise. Those who had the capacity to continue their usual exercise routine found that the PICC prevented usual activities such as swimming. Some of these participants ceased exercise altogether which they felt impacted their overall health. Others replaced swimming with an activity that was safe for the PICC such as walking to try to maintain fitness levels.

‘…but we have a swimming pool which I am really disappointed not swimming at the moment because that keeps my weight down. I usually swim about 600 meters…I used to swim five days a week and do exercises on one day a week in the gym.’ P1

#### Caring for children

Participants with children who were school age or younger were required to modify how they interacted with their child to protect the PICC from accidental dislodgement. This included modifying how they played with their child, especially for those with children that enjoyed physical play, such as wrestling. Those with younger children described how they were required to modify how they lifted and carried their child, which was challenging. They stopped lifting their child to comfort them in order to protect the PICC. Some found that adapting usual parenting activities was the biggest challenge they experienced at home. Challenges in adapting parenting were amplified for sole parents who had minimal support at home.

‘I couldn’t lift them up or do the things I would normally do. They could sit on my lap a little bit, but it changed the way I interacted with them, definitely, for about four or five weeks, all up.’P5

Parents were also required to support children to modify their behaviour to protect the PICC. Educating children about the device was effective with older children who could follow direction, but those with younger children were required to physically protect the PICC from the child. They found over time the child accepted the device and this was no longer necessary.

‘Yeah (the toddler tried) to touch it. Yeah (and pull it out)…they used to, when they see oh you know it’s six months I have a PICC line and they used to it.’ P12

#### Social relationships

Most participants did not modify their usual social interactions due to the PICC. However, many described that they were not socially active anyway due to the risk of COVID-19 or lethargy from their health condition and treatment. Those who continued their usual social life described that the PICC impacted interaction with friends and family. Some found that their friends and family members were wary about damaging the PICC.

‘I think my mother-in-law would often like to give me a hug, but she was very careful about doing that, because she knew I had the PICC line. That would be the biggest things people – I just didn’t hug people, like I would.’P5

Some participants indicated that they modified how they acted when in public places with large groups of people to protect the PICC.

### Physical comfort

Participants described discomfort associated with PICC insertion and due to skin damage related to dressing products and where the external parts of the PICC were positioned.

#### Initial and ongoing discomfort from PICC insertion

While most participants found that both PICC insertion and living with the device was comfortable, some described discomfort during the insertion or immediately following PICC insertion. Although most found their pain resolved over time, some experienced prolonged discomfort from their PICC which affected daily life.

‘Two or three weeks, it was very tender yeah. First couple of nights I couldn’t sleep – without pain killers I wouldn’t have been able to sleep. It was a situation where I couldn’t even raise my arm. So that was a bit of an issue, but it came good, it was only a matter of time. Pretty rare that I think they get those, but I was just unlucky, but after that it worked fine.’ P3

#### (Dis)comfort from the PICC dressing

Some participants described ongoing discomfort from injuries due to external parts of the PICC or the PICC dressing. This appeared more pronounced in those undergoing cancer treatment. Participants who experienced skin injuries due to the needleless connectors described that this was caused by the way nurses angled the PICC during the dressing changes, which meant that the needleless connectors sat in their cubital fossa. One participant described bruising in this location which was exacerbated due to their underlying condition.

‘The only issues I really had was you really had to make sure it was in the right position angle wise, especially on my arm, because if you didn’t have it angled properly it would get caught in the crevice of your elbow at night and it would leave a bruise there in the end… and it gets pretty sore.’ P3.

Their comfort was improved when nurses moved the securement device to angle the external parts of the PICC away from their cubital fossa during the dressing change. Other participants described skin injury or irritation due to the adhesive securement device. They found that their skin under the device became irritated and itchy, which was exacerbated in warmer weather with increased perspiration. One participant identified that they had impaired skin integrity and described that their skin was damaged each time the adhesive securement device was removed during the PICC dressing.

‘… my skin is very fragile and they put one of those sticky things on to hold it in place, …if you stick things on it rips my skin when you take it off.’ P11

### Self-management

Participants described the importance of taking responsibility for the PICC to ensure safety. One participant described self-management as integral to their role as a health consumer undergoing treatment with a PICC. Self-management included not only following instructions provided by clinicians to protect the PICC, but also active participation in their own care. This included developing skills in self-assessment, self-advocacy and managing clinical care.

#### Taking responsibility

All participants identified that it was their responsibility to follow instructions provided by clinicians, such as keeping the dressing dry to reduce the risk of complications. Some also identified that it was their role as an individual undergoing treatment in the community to take increased responsibility for their health and the management of the PICC. This included taking responsibility for identifying PICC complications independently and contacting clinicians when required.

‘Well I think you’ve got to (be) a little bit responsible for yourself. Because I mean the backup is very good but they can’t do everything. You know you’ve got to tell them if there’s a problem.’ P14

#### Self-advocacy and managing care

Several participants identified the importance of developing advocacy skills to question nurses when practice deviated from their experience of clinical care.

‘…we should take responsibility for most things that happen. We’re hopeful that people that come in and do it know exactly what they’re doing, hopefully. I think earlier on when I was at (non-specialist hospital) there was a variety of people that were good at it and some people that were not.’ P11

Participants developed an understanding of PICC clinical care as a hospital in-patient or through visiting the OPD of specialist hospitals. Ensuring that nurses decontaminated the needleless connector for sufficient time prior to connecting an infusion was identified as an aspect of clinical care that required surveillance. Once they experienced a lapse in clinical standards, participants began to monitor the clinical practice of all nurses in order to protect themselves.

‘I just wanted to make sure everyone was doing the job right, because it is – it’s on you, and it’s close to your heart…’ P5

Advocating for themselves and questioning clinical care provided by nurses was initially challenging. Participants developed the resolve and skills over time as they gained knowledge and confidence.

‘I’d say early on in my treatment which now seems a while ago I probably wasn’t as good a patient as I am now. So if there (are) differences in what one person …(is) doing as opposed to another person … I would point it out or I’d feel – I’d question it, I found that a little bit hard initially.’ P11

It was important that questioning nurses about their care was performed diplomatically to ensure a positive ongoing relationship. Self-advocacy was especially challenging for some participants due to treatment side-effects and they relied on their spouse to advocate for them and question clinicians when they felt clinical practice was suboptimal. Some nurses did not react well to health consumers or caregivers questioning their clinical practice. One participant described that they had a skin reaction to usual dressings and required a modified dressing protocol. This meant that they were required to remind the clinical staff visiting their home to change the dressing each time they visited, and some nurses appeared hostile to them advocating for a different dressing.

Once participants had lived with the PICC, some also learnt to advocate during the PICC dressing in order to maximise their comfort. They asked nurses to angle the PICC to keep the needleless connectors away from their cubital fossa to minimise impact on daily life.

‘…say .. well righto, just make sure the bungs (needleless connectors) are up here or whatever… and they’ll curl it around a bit so it’s sort of up more on your bicep rather than down towards the crook of your elbow so it doesn’t affect it.’ P7

Participants also described that they faced challenges navigating non-specialist settings and were required to take initiative to access appropriate care. One participant found such settings used different needleless connectors to the main cancer centre. After several blood tests haemolysed with these needleless connectors, they took responsibility to obtain a supply of needleless connectors to provide non-specialist settings for subsequent dressing changes. Finding trained clinicians to take blood from the PICC at non-specialist pathology collection centres was challenging. Participants learnt to identify when appropriately trained nurses were working to ensure that they could have blood sampling from the PICC to minimise venepuncture. One participant faced difficulty finding nurses trained in PICC dressing changes in the rural area they lived. In order to avoid travelling to the main cancer centre they supported nurses at the local hospital to learn the PICC dressing procedure.

‘I’ve helped them upskill because I’ve taught about four nurses over here what needs to happen with the PICC line dressing, so it’s all good.’ P10

#### Self-assessment of complications

Participants with both infection and cancer reported that they regularly assessed their temperature and the PICC as recommended in the information provided by the health setting. Assessment of the PICC insertion site and external length became a routine part of their daily life. One participant who worried about dislodgement regularly measured the external length, which provided reassurance when they were concerned the PICC had become dislodged.

… if I was starting to get concerned, I’d measure it myself and just go, oh no, that’s right, that’s all good…I’d always check, because I thought, oh, did I just pull it then? Then, instead of panicking and waiting for a whole day before you see a nurse again, I just got my tape measure out … That’s a big thing, knowing – and that reassures you not to worry about it. P5

### Emotional impact

Many participants were concerned about both the PICC insertion procedure and living with a medical device inside their body. Some participants also described worry about their ability to adapt to the device at home and complications that could occur. This concern was heightened as they were required to monitor these complications independently. The emotional impact of the device on friends and family were also important considerations for the individual living with a PICC.

#### Insertion

Participants were apprehensive about the risk of complications and pain during PICC insertion. For some, the consent process and explanation of potential complications that could occur increased this anxiety.

‘To me, the hardest bit of it all was getting it in because they had explained about the dangers of it, which is fine, I mean I would like them to do so. But that means that you’re a little bit tense about getting it in, apart from that I had no worries about it… Well, that was the worst part for me was getting it inserted because I knew the dangers, but you know that’s something you have to go through.’ P1

Participants were worried about whether they would experience pain during the procedure. Their concern increased once they learnt they would not receive sedation during the procedure.

Both participants with cancer and infection described that learning about the need for a PICC was overwhelming as it came at the same time they received their diagnosis and copious information about the treatment plan. For some, the time to PICC insertion was rapid and there was little time to absorb the need to undergo PICC insertion and they described that time as a ‘bit of a blur.’

‘…No I was never prepared for anything. You know as far as a cancer of any sort goes you’re never prepared, and it was an absolute daunting time when they said we’re going to insert a PICC.’ P3

One participant with cancer described that they were apprehensive about each test and procedure they were required to complete for cancer treatment work-up and this influenced their experience of PICC insertion. They recounted that they experienced palpitations during the procedure due to their concern about PICC insertion.

#### Coping at home

Participants who were in-patients when they had the PICC inserted described apprehension about going home with the device when the plan for discharge was raised. They described worry about coping with daily life at home due to perceived increased risk to the PICC when recommencing usual activities. This concern was heightened if they lived alone or had children. One participant who lived alone and was discharged home on OPAT identified that they were worried initially, but after they had experienced life at home with the PICC and a nurse conducted a home visit to check the PICC they felt reassured.

‘I’m now a widow so I’m on my own, so even more concerned when I had the PICC in... I think there is a bit of what would I say, concern until you’ve done the first 24 hours. … I was very glad once the 24, once the nurse had been in once….’ P1

This concern was amplified for those who had caregiver responsibilities at home. One participant, who was a sole parent of a toddler, described that they were concerned about managing by themselves at home and how they would keep the device safe.

‘Well I was a bit nervous because I’d done very little for four weeks and I was really weak and I had a 21 month old and I was told not to lift anything and I’m a single parent.’ P13

#### PICC inside my body

Concern about the PICC sitting inside their body was pronounced for many participants. The device was viewed as a foreign entity inside their body that ‘exposed’ them to the risk of complications. Most participants had a PICC for many weeks, and the length of time they had a PICC in their body increased their concern.

‘Actually I think I was more concerned about having the PICC for six weeks than the chemo.’ P11

The location of the PICC tip was the source of most worry for participants. Information about PICC insertion provided during the consent process, which detailed where the PICC tip would be located, increased their worry. The proximity of the PICC to the heart was identified as anxiety provoking by many participants, as they were concerned it may damage their heart. Others were concerned that the PICC tip location would make complications such as infection more problematic as bacteria could travel directly to their heart.

‘Well I think you know when you sort of think of something going around into your heart – you know into the vessels of your heart or just the sort of where it is I think that made me a bit anxious… you think “Ooh is it safe?’ P14

#### Monitoring complications

Participants with both infection and cancer expressed concern about complications that could occur at home, which was amplified as they were required to identify these independently. Risk of infection, dislodgement and bleeding were all identified as a concern. For many participants, this initial concern about complication risk subsided after they had experienced living with the PICC without a problem. Some stated that the concern about getting the dressing wet in the shower and developing an infection was a continuous concern.

The requirement to independently monitor for complications at home was a source of worry for some participants. Those who were hospitalised were concerned upon discharge home as the PICC would no longer be checked as frequently by clinicians. They were anxious about whether they would be able to correctly assess the PICC to identify a complication, and successfully manage a problem if it occurred. One participant experienced extensive bleeding from her PICC site whilst hospitalised and was worried that would occur again when they were alone at home.

‘Sometimes yeah, yeah (I was worried about monitoring for complications) … If it’s got a problem, because …I don’t know what happened… it just get a bleeding from my veins, it was very horrible… Yeah I just saw it, it was a lot and it was horrible. And they just wrap it in the hospital the nurse and I was very worried that this happen in the house.’ P12

#### Acceptance

Participants indicated that it was important to accept the PICC quickly in order to continue daily life as soon as possible. Once they accepted the need for the device, they were able to start adapting usual activities to continue living their life. Many found that viewing the PICC as a means to enable treatment, which allowed them to live outside of hospital, assisted with accepting the device. Others described the importance of a positive attitude and that viewing the PICC as better than the alternative of undergoing multiple cannula insertions helped them to accept the PICC.

‘Well look I sort of think things are what they are and you know if this is going to help let’s do it. That’s my approach. Yeah so I felt lucky that in many respects that there were treatments that could actually assist. So I tried to look at it in that respect and not focus too much on the negatives.’ P14

The time to acceptance of the device differed for participants. Some immediately accepted that they required a PICC as soon as it was raised by their medical team, while others only accepted the device after living with it for many weeks.

#### Those around me

Participants also described the emotional impact of the PICC on family members. For some participants with cancer, the device came to signify the disease itself, as it was viewed as the only visible manifestation of their health condition. Participants thought that family members were initially uneasy and concerned about the PICC. Some friends and family members found the PICC confronting, and participants indicated they felt they should have prepared their family for the PICC to reduce their worry.

Participants with young children reported that it was important to involve the child in PICC care and educate them about modifications required to their usual interactions in order to reduce their worry and fear. Parents who were unable to continue lifting their child to comfort them, recounted that it was important that the child understood that this was necessary to protect the PICC, and the parent was not rejecting them. One participant initially hid the PICC from their toddler to shield them from the cancer diagnosis. Upon advice from a mental health specialist, the participant showed their child the PICC and allowed them to watch dressing changes. They also read picture books with their child about hospitals which contained people with bandages to explain that their bandage (PICC cover/dressing) would help make ‘Mummy better.’ It was important to normalise the PICC and the PICC dressing to reduce their child’s fear and improve their connection.

‘…I don’t want to be like a fragile thing that he’s scared of being near. I’ve chosen to involve him in stuff. Like I said he’s often here. So he often talks about it and looks at it.’ P13

### Information content

Participants indicated that they required further clinical and practical information about the PICC. Most information provided to participants focussed on risk, either during the insertion procedure or catheter-related complications that could occur with the device at home. While participants understood that this information was important, they also required information about the experience and duration of the insertion procedure and practical information to support them to adapt at home. Information to support self-management, including recognising and responding to PICC complications was also identified as an information need.

#### Clinical information

Participants described that they received minimal information about the PICC insertion process. Many described that the only information they received was verbal and delivered by the PICC inserter just prior to the procedure. While they found these clinicians knowledgeable and their explanation reduced their concerns, they indicated that receiving this explanation during initial treatment planning would be useful to reduce worry.

Participants were unsure if they would receive sedation or if the procedure would be painful. Other participants wanted to understand the reasoning behind arm choice for insertion and why PICC inserters favoured the right arm (which for most was their dominant arm). The provision of further information about the method used by PICC inserters to verify tip location was important to reduce fear.

‘But I think – like you know with a PICC line perhaps a little bit of sort of education before it goes in. You know what to expect. Because it’s not a painful process. I mean they’re very skilled at what they do. And – but it’s just the anxiety of sort of having something inserted and you know and you know it’s at the top of your (heart)... So you know just all that is a bit you know anxiety provoking I think. And you do worry what could go wrong.’ P14

Some participants identified that they were information seekers who typically researched every medical procedure they underwent. They recounted that they used a video sharing platform to access further information about PICC insertion and found that these videos were outdated or did not reflect the products used at their health setting. However, this research did enable them to understand the insertion process, although for some, watching videos of the insertion process online increased their worry. It was important that a credible source of information was accessed. However, many found a lack of local health department or government sites and they indicated that individuals should appraise the source of the video.

‘You know, you’ve got to be able to read between the lines and say yeah well we won’t go with that.’ P7

Participants indicated that after they experienced PICC insertion, they were surprised that the procedure took little time and thought that the provision of this information to other consumers would be useful prior to the procedure to reduce anxiety.

Further information to support individuals to take responsibility for the device was identified as essential by participants. Some indicated that clinicians should clearly inform consumers that they were required to take responsibility for the PICC at home to make their role clear. Further information about self-assessment of PICC complications and decision-making when a problem occurred was identified as important to ensure safety. Some participants were unsure about differentiating between a complication and expected outcome, especially when they experienced pain around the insertion site.

‘And sometimes you can just sort of sleep in a funny angle or you know you’re a bit stiff when you wake up in the morning so you know but you’re aware of – you know particularly sort of I think they said shoulders and anything around the sort of PICC line where it – yeah so I think a little bit more education with that would be – would help people to relax a bit I think.’ P14

While most participants identified that they wanted accurate information about PICC complications, some thought that the focus on adverse events in the PICC information sheet increased their anxiety. They thought it would be beneficial to highlight that most people with a PICC do not experience a complication in PICC information.

‘I don’t want to have anything hidden from me and my doctor said, “I’ll always tell you the truth. We don’t hide things from you”. I think that is easier for you to accept what’s happening when you’re being told the truth than people sort of sugar coating the pill. But on the other hand you don’t want – in terms of the PICC, you know, you don’t want kind of scare people who don’t know what’s happening into thinking, “Oh, it’s terrible”. Because I’m sure there are lots and lots of people who don’t find it an issue at all.’ P2

Participants with cancer indicated that information about PICC complications centred on infection and some suggested that it would also be helpful to include information about other complications such as dislodgement. One participant identified that it was important to provide information about bleeding, specifically, when to seek a dressing change in-between scheduled dressing changes.

Participants identified that further information about the PICC removal process should be provided. They were uncertain if they were required to attend the Radiology Department to undergo another procedure or whether removal would be painful. One participant who indicated that they were anxious about pain they may experience during removal, found the process comfortable and identified that further information provided before PICC removal may reduce their worry.

#### Practical information

Practical information needs regarding showering, sleeping, childcare and exercise were clearly stated by participants. One participant identified that they were unsure about sexual positions that were safe with a PICC and that clinicians had never broached this topic with them. Practical information about how to live with a PICC was important, especially specific instruction on how to shower safely.

‘…how to live with a PICC line, you know; to keep it dry and all that sort of stuff. Yeah. So I think, yeah, probably a little bit more information or guidance on how to live with a PICC line I guess.’ P6

Some participants indicated that it would be useful if clinicians assessed their responsibilities at home to assist them to develop safe strategies to continue their usual roles. One participant who was a sole parent wanted initial information about how to continue usual life with their toddler. This included how to lift a toddler who was over the weight limit suggested for those with a PICC and bathe the child. Other participants required further information about how to exercise safely with a PICC. Many participants with cancer were aware that exercise was recommended when undergoing treatment, but were unsure how to modify activities to protect the PICC.

‘They talk about the importance of exercise…But then I have thought well how am I going to do … yoga or whatever with this because lots of that stuff I think would not really be recommended with this. So I reckon that’s an area with this – it probably has inhibited me a bit from getting into doing anything because I think oh well how am I going to do it with my PICC?’ P13

### Understanding information

Participants described varied understanding of the information provided about the PICC. While many found the information easy to understand, they indicated that they were unsure if other people would understand this information due to the impact of age and co-morbid conditions. The medical terminology used by clinicians made understanding information more challenging. Timing also reduced their understanding as participants indicated that they were overwhelmed by treatment and diagnosis information when they received information about the PICC. Participants reported varied preferences for the format used to provide information about the device.

#### Format

Many participants indicated that they found individual one-on-one information provision provided by a nurse was useful to support them to understand information, as they felt comfortable asking questions. Other participants indicated that they preferred information to be provided in a combination of formats (online information, group education, one on one with a nurse). Peer discussion forums with other individuals who had experienced living with a PICC were also identified as beneficial to improve information comprehension. Some preferred PICC information to be provided online; it was important that this was produced by a reputable source such as a health department.

Someone like me would go online first, and educational videos put out by the (hospital) or government … click here to see how a PICC line is inserted, like, watch how to shower, even if it’s on a dummy, just showing them how to pull the sleeve on. … I think making sure that – just videos on, I don’t know, what it looks like, before you get it, would be – that’s how I would look online, that’s what I would look for online. P5

Many participants thought that paper-based information was important for those with limited access to the internet or those who were uncomfortable using computers.

#### Language style

Some participants found that information about PICC insertion was difficult to understand due to medical jargon, which increased their concerns about the insertion. They identified that information should be written in simple language to aid understanding in order to reduce the concerns of people undergoing PICC insertion. Simple language was especially important for those with a cancer diagnosis who received PICC information on top of a large amount of information about treatment.

‘I kind of think sometimes if it’s too medically oriented people don’t understand always and, you know, I think that makes it a little bit more scary … you know, written in ordinary English not too medically oriented… because I think some people probably panic and see the worst when there doesn’t have to be a worst.’ P2

#### Timing and amount of information

Participants found that PICC information was not provided at an appropriate time. Earlier provision of PICC information was thought to reduce worry about insertion and improve understanding of how to live with a PICC. Some indicated it would be useful for the PICC inserter to attend the treatment planning meeting with the medical team when the PICC was first raised as an option, so that they could address their concerns about insertion.

Some participants described that their information needs changed over time. They did not want detailed PICC information initially as they felt overwhelmed by treatment information. They described that after they had become comfortable with their diagnosis and treatment plan, many felt ready to absorb further information about the PICC. Others described that they forgot information, and it would be beneficial for nurses to repeat information at the weekly dressing change.

Needs regarding the amount of information differed. Some participants wanted as much information as possible and others preferred minimal information. They indicated that clinicians should vary the amount of information provided based on individual preferences. One participant thought that providing too much information, especially about PICC complications, increased distress as some people would worry about potential adverse events.

‘…sometimes you can give people too much information and confuse the issue. It’s the devil or the deep blue sea… Some people just need to know that it works and don’t want to know the rest of it... You can tell people a whole lot of stuff and they go away and worry themselves to death because they’ve got all this overload of information, compared to … as long as I’m careful with it… I keep it … Keep it simple and be done with it……some people don’t want to know… But others want to know a damn sight more.’ P7

Some participants felt overwhelmed with the information provided about the PICC when it coincided with diagnosis. This was most obvious with those with cancer who felt overwhelmed by the substantial information provided about diagnosis and treatment and had learnt to ‘deal with’ their condition by avoiding detailed information. Hence, the amount of information provided at that time was viewed as challenging and they thought that PICC information should be minimal and practical.

‘To be honest I think, you know, when you’ve been diagnosed with cancer and that’s the reason why you’ve got your PICC in, I mean, you’re already – your head’s already going at a million miles an hour thinking about that. So I guess sort of the more simple and easy it is the more, I guess the more comfort people can take from that.’ P3

#### Including caregivers

Participants indicated that clinicians should ensure that caregivers are present during PICC education. Providing PICC education for caregivers was important so they could support health consumers in their understanding of PICC management and also to assist caregivers to adapt to their role.

‘…(it) would be probably a good idea, if someone was caring for you, that they were instructed, also, on how to – the things with caring for and looking for issues with the PICC line. That would be something that might be handy for the carer to know, but the nurses knew, the community care nurses know about them, but your partner or the in-house carer, if they don’t know about it, it’s a new thing for them too, to live with.’ P5

Caregivers were viewed as a ‘second set of ears’ during interactions with clinicians including PICC education. Some participants described that their brain became ‘foggy’ due to their condition, and it was imperative that caregivers received and understood information about PICC management. This included monitoring for complications, as they were unable to retain this information.

Social supports were needed to provide both practical and emotional support when undergoing treatment with a PICC.

### Healthcare resources

Healthcare supports included healthcare products to protect the PICC as well as support from clinicians. Initial and ongoing support from nurses were important to promote adaptation, support individuals to take responsibility for the device at home, and to quell concerns about the PICC.

#### Healthcare products

The shower covers provided by the health setting were perceived as valuable by many participants, in order to keep the dressing dry and intact to prevent infection. However, some participants found that the shower cover was ineffective. They described that the shower covers often leaked, and they learnt to use multiple covers in the shower to ensure that it worked.

‘…you know, you have the sleeve but those sleeves tend to leak if you get them wet and water runs off your head and that sort of thing and down your arm and the next thing you know it’s leaked through.’ P7

One participant suggested that a new design that kept the PICC dressing dry was required for people living at home with a PICC. Other participants stopped using the covers and used commercial plastic wrap which they found more effective.

All participants were provided with a bamboo PICC cover which they found beneficial to keep the external parts of the PICC covered and secure. For those who had previously experienced treatment with an elastic bandage to cover their PICC, the new PICC cover was perceived as superior.

‘…now they’ve gone to (bamboo cover), it’s a whole different ballgame, they’re a lot more comfortable to wear, and yeah, it just makes life so much easier.’ P7

The PICC cover hid the PICC from other people and it also reduced the risk of dislodgement. One participant with a toddler described that their child would often try to pull the external length of the PICC. The PICC cover protected these external parts, which allowed them to continue family life as normally as possible while keeping the PICC safe.

#### Clinician supports

Specialist nursing staff who managed care were described as ‘impressive’ and support from these clinicians enabled participants to feel safe. Regular contact with nurses (whether at the OPD or home) during the infusion or dressing change allowed participants to seek clarification when they were unsure.

Participants indicated that contact with nurses reduced their worry about the PICC. Regular contact with these clinicians was especially important for those participants undergoing treatment at home. The first home visit and check of the PICC by the nurse was identified as a turning point for participants. Once this had been completed, they felt at ease with the device and undergoing treatment at home. Ongoing contact with nurses during weekly dressings was important to ensure that their PICC was functioning without any complications, which reduced their concern about the device.

‘Because every week your PICC line is cleaned and checked, that relieves the worry because it’s measured every time…that’s reassuring.’ P10

Both nurses at the infusion centre and those who visited the home to change the infusion were perceived as important resources to support participants to take responsibility to manage their device. This support was essential to enhance coping and ease the transition to self-management. Access to telephone support from these specialist centres was also important to ease worry about complications. Many participants had their contact details still readily accessible at the time of interview.

‘I mean just in general that was a massive change because it was so heavily monitored in hospital so highly monitored and then suddenly I was going home. … I think that was – if I didn’t have that (clinician phone numbers) it would be a completely different story. ‘P13

Participants identified that they required further support from healthcare settings and clinicians to assist them to modify showering at home. Participants identified that it would be useful for nurses to discuss the bathroom arrangement of those about to be discharged home and develop strategies to support them to adapt showering. One participant suggested that nurses could attend the home of people undergoing OPAT, especially those who lived alone, to ensure that the bathroom setup was appropriate to allow them to easily adapt to showering with a PICC.

‘I think it would really help for some women on their own and some men, to have a nurse come home with them and check them out at home… it’s really important to discuss somebody going home what their shower and bath arrangements are….That really does need to be a bit more use and discussion …I think that’s the main thing that really needs to be looked at is what is your situation at home in the bathroom.’ P1

#### Healthcare systems

Participants required the health care system to provide coordinated care with appropriately trained nurses at all healthcare settings. This was more prominent for those with cancer who described challenges with accessing skilled clinicians away from the specialist centre, such as pathology centres and ‘non-specialist’ health settings. Some participants living in rural areas faced difficulty in accessing adequately trained nurses to take blood from the PICC and change the dressing. Participants identified that it would be useful if all healthcare settings, including pathology centres, were staffed by trained nurses who could obtain blood samples from the device.

‘Well they will not use the PICC. I think some of them are trained to do the PICC and some are not trained to do the PICC in terms of the pathology people. So that would’ve been really handy for them to just come in and (obtain a blood sample) …– yeah, that would be helpful, very helpful.’ P11

### Social supports

Social supports were viewed as essential when undergoing treatment with a PICC. Family and friends were required to provide both practical and emotional support.

#### Social resources

Social support was important to enable participants to adapt to life with a PICC. Life partners/ spouses were integral to continue daily life and ensure safety for many participants. Participants described that they managed the PICC together with their spouse who provided both practical supports and emotional comfort. Friends and neighbours also provided support to many participants through regular visits and assistance with tasks.

‘I thought I was pretty covered because I had my husband here who’s a fantastic support and, yeah, he knew the situation was pretty, you know, all – if something does go wrong, you know, you’ve really got to get in there and get her sorted. So he was all over it and, yeah, had a lot of support around me as well with neighbours dropping in and checking on me and stuff.’ P6

#### Knowledgeable caregivers

It was important that caregivers understood clinical information about the PICC to provide appropriate support. Some participants were supported by family members who were nurses or physicians. They described that their caregiver was able to translate medical information and provide practical support such as applying the PICC shower cover correctly to keep the PICC dressing dry in the shower. Their existing clinical knowledge was useful to provide an additional check to ensure that the PICC was functioning. Other participants described that while their caregiver did not have a clinical background, their knowledge increased over time, which was an invaluable support.

## Discussion

The study aimed to identify the supportive care needs of people living with a PICC at home through individual interviews with 15 health consumers. A diverse range of supportive care needs were identified by participants in the following domains: adapting daily life, physical comfort, self-management, emotional impact, information content, understanding information, healthcare resources, and social supports. Some needs were shared by all participants, while others depended on personal circumstances such as caring responsibilities. These findings provide nurses and other clinicians an indication of the challenges and needs of individuals living with a PICC at home. This may provide a framework to guide needs assessment, education, and indicate interventions that may assist health consumers to assume an active role in adapting to life with a PICC.

Adapting daily life at home, such as showering, was the most common supportive care need stated. Most participants faced practical challenges, especially adapting showering to keep the PICC dressing dry in the shower. Many of the practical challenges and supports required to adapt daily life support previous research that has documented the experience of people with a PICC [9, 10, 12, 13, 15, 19]. Participants also raised other practical challenges they faced, such as modifying exercise, parenting, and sexual relationships which have not been identified previously. Health consumers with a PICC have individual needs and the assessment of their needs and the provision of support to adapt to these practical challenges is imperative to ensure that they maintain function and safely participate in this model of care.

Support from both healthcare settings and social networks were identified as important to aid adaptation. Participants explained how their family and friends acted as their carer and advocate while they were receiving treatment in the community and learning to live with a PICC. The important role that caregivers play is recognised in cancer treatment research. In comparison, the caregiver’s role in OPAT is less reported [8, 39]. Our study findings demonstrate that caregivers play an equally important role in assisting health consumers with cancer and infection in the community. Including caregivers in clinical education and decision making ensures that they have access to a social support network during their treatment journey.

Participants described emotional needs both for themselves and for those around them. Clinical aspects of the device such as insertion and risk of adverse events at home induced anxiety. Fear due to the location of the tip ‘*near the heart’* was pronounced. Concern resulting from the location of the PICC tip has been identified previously [9, 12]. Clinicians understand the benefits of the tip location which provides greater haemodilution to allow the infusion of irritating solutions [40]. Further education about the rationale for the tip location may reduce worry for individuals booked for PICC insertion. Participants also reported concerns about their ability to cope at home with a PICC, and the impact of the device on their family, especially for those with young children. Parents were required to modify interactions and to support children to accept the device to reduce their fear. Many of these participants developed strategies to support their children independently. To many clinicians, a PICC is part of routine care, a means to enable treatment for cancer or infection. They may underestimate the health consumer experience and stressors of insertion and living with the device. Research in the consumer experience of other vascular access devices in children and adults has found that both insertion and living with a device may be distressing and the psychological impact may not be appreciated by clinicians. [17, 41–44].

Practical information needs were evident in the present study. Many participants described that they required further information to support adaptation of daily activities at home. Most information concentrated on risk of complications, either at insertion or when living at home. While they appreciated that it was important to receive this information, they also required information to assist them to adapt usual activities such as showering, sleeping, childcare, exercise and intimate relationships. This aligns with previous research which found that individuals with a PICC found information about living with the device was minimal and they were not prepared for life with a PICC [15]. Participants in the present study identified that it would be useful if clinicians assessed their responsibilities at home and assisted them to develop strategies to allow them to continue their usual role.

The findings of this study add to the literature by proposing that self-management is a supportive care need of individuals with a PICC. While supportive care need frameworks such as Fitch [27] are focused on empowering health consumers, they do not include a specific domain relating to the supports required by individuals to undertake an active self-management role. Health consumers have reported in previous research that taking responsibility for the device at home was onerous and made them uneasy [13]. Conversely, in the present study, some study participants readily accepted this role, but required further support to undertake this task. Self-management involved active participation in their own care such as developing skills in self-assessment, self-advocacy and managing clinical care. Some participants took an educator role, instructing inexperienced nurses to change the PICC dressing and measure the external length correctly. A growing body of research has examined interventions to support self-management or ‘patient activation’ which is the individual’s knowledge, skills and confidence to self-manage their health [45]. Such interventions have been shown to improve clinical outcomes such as reduced glycated haemoglobin (HbA1C) in adults with diabetes mellitus [46]. An ‘activated’ health consumer is crucial for safe and quality PICC management at home, as they are required to identify and triage complications and navigate the health system to access appropriate care, which as we saw in this study was often challenging, especially in non-specialist treatment centres. In developing interventions aimed to empower or ‘activate’ individuals with a PICC, it is important to note that this requires more than the provision of appropriate information. It also requires clinicians to support individuals to manage their own health including clarifying their role in their own healthcare.

To our knowledge, this is the first study to investigate the supportive care needs of adults with a PICC. These findings provide nurses and other clinicians with an understanding of the challenges and supports required by individuals living with a PICC at home. This may guide clinical assessment and education to support individuals to adapt to life with a PICC. The study findings indicate that adults with a PICC may have unmet needs. It is important clinicians are able to assess these needs to provide appropriate supports to improve adaptation and reduce their concerns. Yet there are no supportive care needs assessment tools that are specific for individuals with a PICC. The assessment of supportive care needs is common in the treatment and management of solid tumours and haematological malignancies. Tools such as the Supportive Care Needs Survey [47] and the National Comprehensive Cancer Network (NCCN) distress thermometer are commonly used in clinical practice to assess generic supportive care needs [48]. The development of a tool to assess the specific needs of adults living with a PICC at home would be valuable to prompt individuals to identify their own needs, make those needs visible to clinicians and guide discussion about supports required [49].

Both participants with cancer and infection in the present study identified needs across the supportive care needs domains, which indicates that a single PICC associated supportive care needs assessment tool may be appropriate. However, needs might differ according to individual characteristics such as residential location, diagnosis, and age/life stage. As we saw in the present study, a wide range of age groups undergo treatment in the community with a PICC and individuals may have different needs based on caring and employment responsibilities. The location of the health consumer may also impact their needs, with people living in rural areas facing different challenges to their counterparts in metropolitan areas. Underlying diagnosis may also impact the needs of adults with a PICC. For example, in the present study, it appeared that participants with a cancer diagnosis were more prone to skin injuries compared to those with an infection. Although not explored in this study, exploring specific needs associated with different treatment regimens (e.g. elastomeric device versus infusions at a clinic) would also be useful. An international survey is recommended to fully elucidate the needs of individuals with a PICC across health settings. This survey will allow the identification of further supportive care needs and importantly, an understanding of how needs may vary according to individual characteristics. This study design would also support the development of a PICC supportive care needs assessment tool to be used in nursing practice. This would allow nurses to assess the supportive care needs of adults living with a PICC to enhance the consumer experience and provide person centred care.

### Limitations

Participants were recruited from one health service and their experience may reflect the practices of that service. Whilst robust qualitative methods were used, a relatively small number of participants took part in the study and consistent with qualitative approaches, findings cannot be generalised to other settings.

## Conclusions

Adults living with a PICC at home report a broad range of supportive care needs. Supporting individuals to adapt daily life and fulfill their roles and responsibilities at home is imperative to maintain functioning and enhance quality of life. Nurses provide valuable assistance to support adaptation to life with a PICC practically and emotionally. The personal circumstances and responsibilities of these individuals outside of healthcare are important considerations when providing education and support for those living with a PICC. An empowered health consumer is integral for safe and quality PICC care at home and supports are required to assist individuals to undertake this role.

### Supplementary Information

**Additional file 1.**

## Data Availability

Data is available from the corresponding author on reasonable request.
