# Supplementary material for: Supportive care needs of adults living with a peripherally inserted central catheter (PICC) at home: a qualitative content analysis
Source: BMC Nurs. 2024 Jan 2;23:4. doi: 10.1186/s12912-023-01614-0 (PMC10759691; doi:10.1186/s12912-023-01614-0)
Supplement: Supplementary file 1 — Additional file 1. [file 12912_2023_1614_MOESM1_ESM.docx]

| **Meaning unit** | **Condensed meaning unit**  **Description close to the text** | **Code** | **Sub-category** | **Category** |
| --- | --- | --- | --- | --- |
| Yeah, it was uncomfortable. They put a local anaesthetic on it, but you can sort of feel it. (15) | Pain during insertion despite anaesthetic | Insertion pain | Initial and ongoing discomfort from PICC insertion | **Physical**  **comfort** |
| I did have a problem with my second one they put in this time, last year they must have hit – not hit something, there was some sort of intrusion on the way in and they had to put it out a little bit and just bruise some internal muscular tissue there. That was very sore but very rare (3) | Pain during insertion | Insertion pain | Initial and ongoing discomfort from PICC insertion |  |
| it was a bit painful at first so it bothered me a bit (14) | Pain immediately after PICC insertion | Initial pain after insertion | Initial and ongoing discomfort from PICC insertion |  |
| No. I mean, it was probably a little bit tender there, but nothing, you know, I’m pretty stoic in a way, so what hurts some, it doesn’t hurt me. (9) | Pain immediately after PICC insertion | Initial pain after insertion | Initial and ongoing discomfort from PICC insertion |  |
| Two or three weeks, it was very tender yeah. First couple of nights I couldn’t sleep – without pain killers I wouldn’t have been able to sleep. It was a situation where I couldn’t even raise my arm. So that was a bit of an issue, but it came good, it was only a matter of time. Pretty rare that I think they get those, but I was just unlucky, but after that it worked fine. (3) | Pain for weeks which interrupted daily life. | Initial pain after insertion | Initial and ongoing discomfort from PICC insertion |  |
| I don't know what's going to happen with this one that’s in at the moment, it's still a little bit painful which is something I haven't encountered with any of the others, the previous ones, but it's just – you know, it just feels like it's bruised like, you know, and you get a corky or something and it's got that numb sort of dead leg feeling. I’ve had a couple of issues with it, it's still sore (7) | Uncertainty about device longevity due to ongoing pain from the PICC | Ongoing pain | Initial and ongoing discomfort from PICC insertion |  |
| and I found it quite uncomfortable. It was taped so that these bits were coming here over my joint so that I couldn't bend my arm really. (13) | PICC dressing can cause discomfort/inhibit arm movement | The way nurses redress the PICC impacts comfort | (Dis)comfort from the PICC dressing |  |
| It's only subsequent to being here that one time someone twisted them around here and went like that and it's been so much better (13) | Nurse changed angle of dressing moved adhesive dressing and changed the angle and it was easier to bend arm/ complete ADLs/comfort | The way nurses redress the PICC impacts comfort | (Dis)comfort from the PICC dressing |  |
| I think that’s the most important thing is you get your lumens on the right angle and after that you don’t even know that they’re there. (3) | Angle makes all the difference  Keep the lumen angled out to prevent pressure injury | The way nurses redress the PICC impacts comfort | (Dis)comfort from the PICC dressing |  |
| … my skin is very fragile and they put one of those sticky things on to hold it in place, …if you stick things on it rips my skin when you take it off (11) | Skin damage from the PICC dressing | PICC dressing products can damage skin | (Dis)comfort from the PICC dressing |  |
| There was one in particular where for some reason these two bits here they sort of oppose like that but there was a bit of a gap and then I got really irritated in there and my skin got all sort of irritated and stuff and they had to redress it when I came in. So some dressings have been a little bit more irritating then others for some reason. (13) | skin reactions from the dressing | PICC dressing products can damage skin | (Dis)comfort from the PICC dressing |  |
| only in the last couple of weeks since its gotten colder and I was wearing more clothes it started to get a little bit rashy underneath the lock - the stat lock or whatever it is. That was a getting a little bit rashy under there, but it was purely because I was wearing more clothes so it was over, you know, it was heating up a little bit more. Yeah. Yeah. That was the only one thing I found. (6) | skin reactions from the dressing | PICC dressing products can damage skin | (Dis)comfort from the PICC dressing |  |
| Probably started off a little bit itchy, if your skin’s got allergies to some sort of tape or whatever it can be itchy yeah. (3) | skin reactions from the dressing | PICC dressing products can damage skin | (Dis)comfort from the PICC dressing |  |
